# The complete mitochondrial genome of the deep-sea paskentanid snail *Alviniconcha marisindica* (Caenogastropoda: abyssochrysoidea) from the Carlsberg Ridge

**DOI:** 10.1080/23802359.2023.2298090

**Published:** 2024-01-03

**Authors:** Mei Yang, Hongliang Wang, Wei Gao, Zhinbin Gan, Xinzheng Li

**Affiliations:** a Department of Marine Organism Taxonomy and Phylogeny, Institute of Oceanology, Chinese Academy of Sciences, Qingdao, China; b Center for Ocean Mega-Science, Chinese Academy of Sciences, Qingdao, China; c National Deep Sea Center, Qingdao, China; d University of Chinese Academy of Sciences, Beijing, China; e Laboratory for Marine Biology and Biotechnology, Qingdao National Laboratory for Marine Science and Technology, Qingdao, China

**Keywords:** *Alviniconcha marisindica*, mitogenome, Abyssochrysoidea, hydrothermal vent, Carlsberg Ridge

## Abstract

*Alviniconcha marisindica* Okutani 2014 is a deep-sea gastropod inhabited in hydrothermal vents of the Indo-Pacific. It belongs to superfamily Abyssochrysoidea. In the present study, we report the complete mitochondrial genome of *A. marisindica*, which is 15,979 bp in length and contains 13 protein-coding genes, 2 rRNA genes, 22 tRNA genes. The nucleotide composition is 29.19% of A, 38.22% of T, 16.88% of G, and 15.71% of C. The phylogenetic analysis indicates that *A. marisindica* and *A. boucheti* clustered in the Abyssochrysoidea clade with high bootstrap support. The mitochondrial genome of *A. marisindica* provides valuable molecular data for further research on the evolution of deep-sea gastropods.

## Introduction

The paskentanid snails *Alviniconcha* (Gastropoda: Abyssochrysoidea) are emblematic inhabitants of Indo-Pacific deep-sea hydrothermal vents, are characterized by a globose and elastic shell decorated with regularly spirally arranged periostracal bristles (Okutani and Ohta 1988; Johnson et al. 2015; Breusing et al. 2020). And most *Alviniconcha* species have symbiotic chemosynthetic Gammaproteobacteria in the gills tissue (Suzuki et al. 2006; Breusing et al. 2022). This genus was erected in 1988 and six species of *Alviniconcha* have been identified and described to date (Okutani and Ohta 1988; Johnson et al. 2015; Laming et al. 2020; Castel et al. 2022). In the present study, we determined the complete mitogenome of *Alviniconcha marisindica* Okutani 2014, which is the second representative mitogenome of the *Alviniconcha* genus (Lee et al. 2020). This research will enrich the taxon sampling of mitogenomes in Abyssochrysoidea and could provide useful molecular data for better understand the phylogeny of deep-sea gastropods.

## Materials and methods

**Figure 1.**
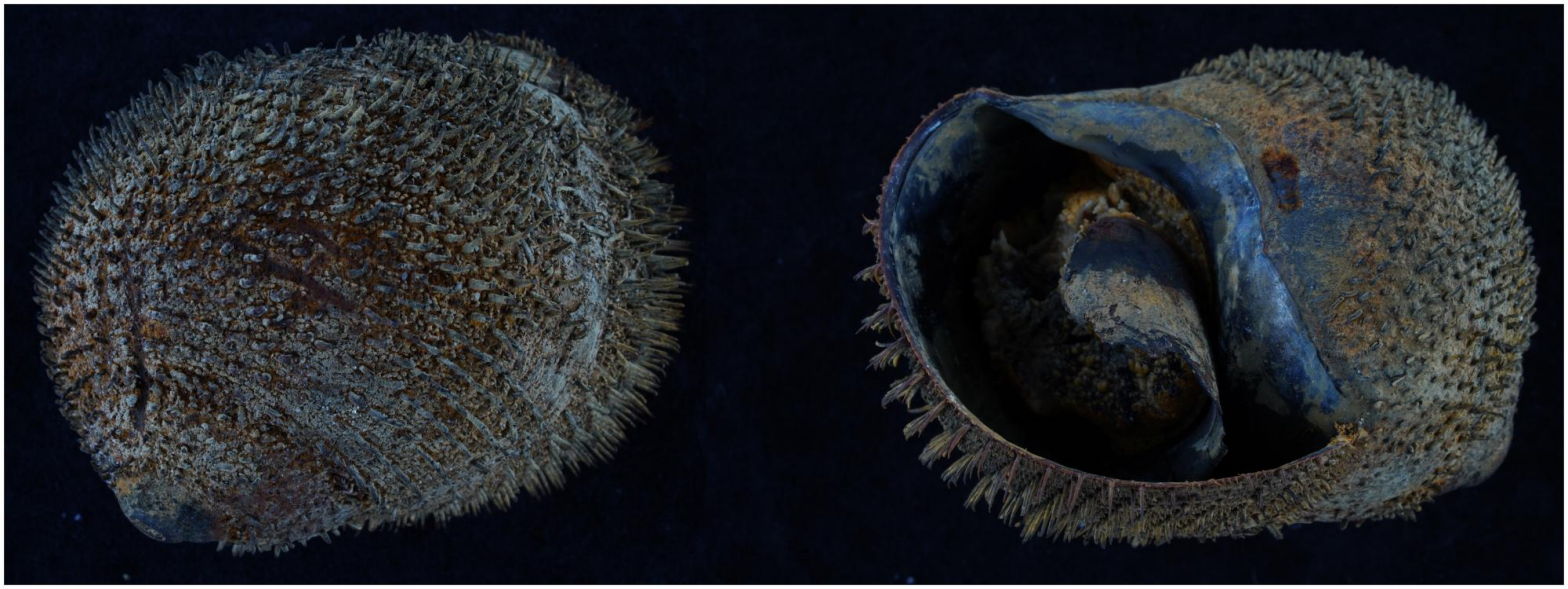
Photographs of *A. marisindica* (photo by Zhibin Gan).

The *A. marisindica* specimen was collected from a hydrothermal vent in the Indian Ocean (3°41′ N, 63°49′ E, depth 3,358 m) in June 2022 by the deep-sea manned submersible Jiaolong (Figure 1). The specimen has been deposited in Marine Biological Museum (Mei Yang, yangmei@qdio.ac.cn), Institute of Oceanology, Chinese Academy of Sciences, under the voucher number: MBM287616.

Genomic DNA was extracted using the DNeasy Tissue Kit (Qiagen, Beijing, China). The paired-end library was obtained using TruSeq^TM^ Nano DNA Sample Prep Kit (Illumina, USA) with an average insert size of 450 bp. Then, the library was sequenced on the Illumina HiSeq 4000 platform with 150 bp paired-end reads length. A total of 35,434,872 clean reads were obtained by Illumina HiSeq sequencing. The mitogenome of *A. marisindica* was assembled by MitoZ v2.3 (Meng et al. 2019) and then annotated by the MITOS2 webserve (Bernt et al. 2013). Finally, the start/stop codons of each protein-coding gene (PCG) were manually corrected in SnapGene Viewer by referencing the mitochondrial genome of *A. boucheti* (NC_049893).

The phylogenetic analyses were conducted based on 13 PCGs of *A. marisindica* and other 24 gastropods from six main lineages, including Caenogastropoda, Heterobranchia, Neomphalina, Neritimorpha, Patellogastropoda and Vetigastropoda. *Octopus bimaculatus* and *Octopus vulgaris* were used as outgroups. The nucleotide sequences of the 13 PCGs were aligned by MAFFT (Katoh et al. 2019). Then phylogenetic relationships were inferred by maximum likelihood (ML) method with the GTR + F + R5 model by using IQ-TREE (Nguyen et al. 2015). The reliability of the tree topology was evaluated using bootstrap support with 1000 replicates. Finally, the phylogenetic trees and node labels were visualized using iTOL (Letunic and Bork 2019).

## Results and discussion

**Figure 2.**
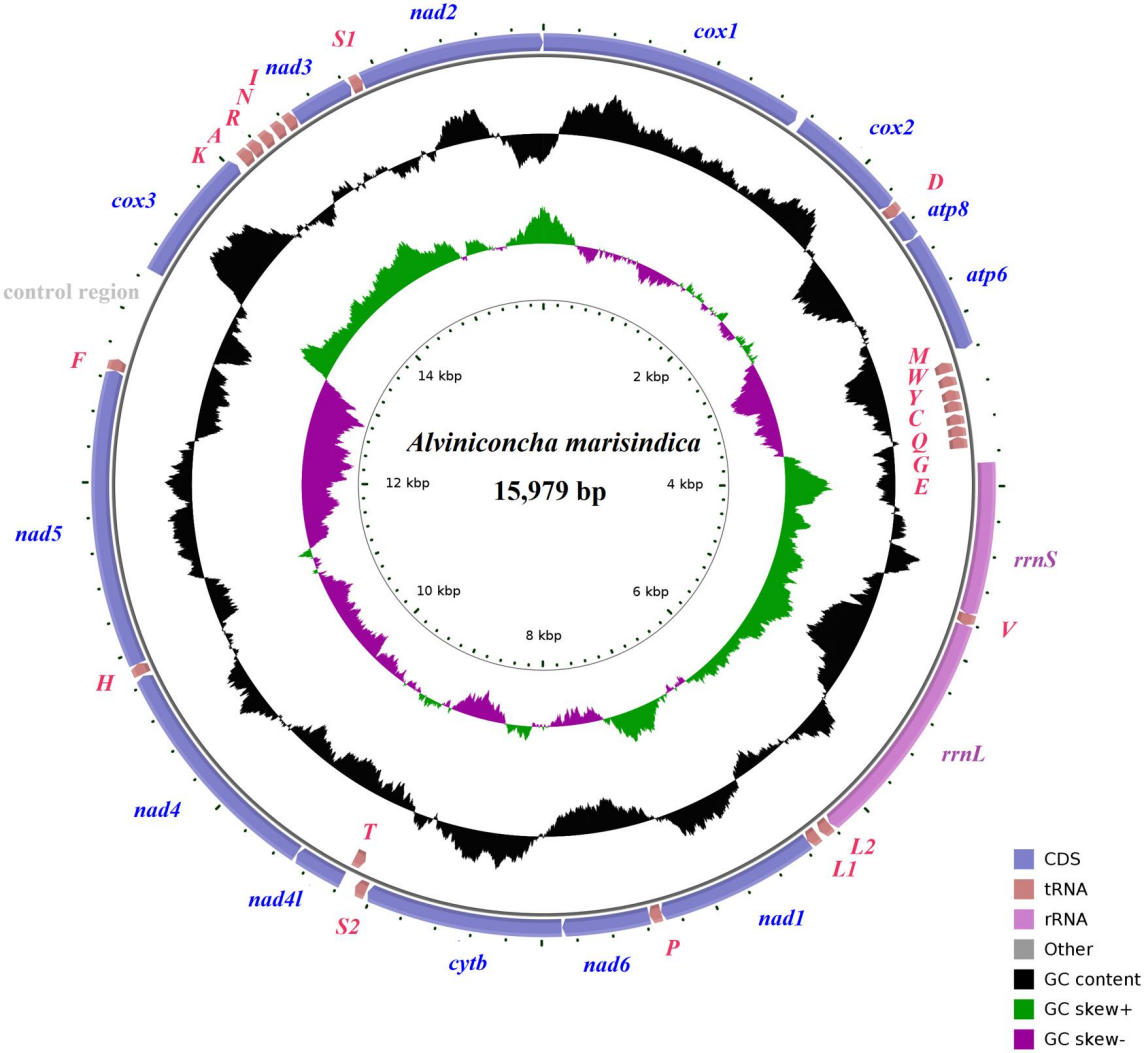
The organization of the mitogenome of *A. marisindica*. Genes for proteins and rRNAs are shown with standard abbreviations. Genes for tRNAs are represented by a single letter for the corresponding amino acid, with two leucine tRNAs and two serine tRNAs differentiated by numerals.

The complete mitochondrial genome of *A.marisindica* is 15,979 bp in length (GenBank accession number: OQ695489) and contains the typical set of 13 PCGs, 2 rRNA genes, 22 tRNA genes (Figure 2). The largest noncoding region (540 bp) located between *trnF* and *cox3* is identified as the putative control region (CR) due to its location in the mitochondrial genome (Lee et al. 2020). Twenty-nine genes were transcribed on the heavy (H) strand, while the others were oriented on the light (L) strand. The nucleotide composition of the mitogenome was significantly AT biased (A 29.19%, T 38.22%, G 16.88%, C 15.71%). The AT skew and GC skew of the genome were −0.134 and 0.036, respectively. The start codon of 11 PCGs was ATG, except ATA for *nad3* and ATT for *nad4*. For stop codon usage, *nad1* and *nad5* stop with the incomplete stop codon (TA- and T–), while the other PCGs terminate with TAA/TAG. Two ribosomal RNA genes (*rrnS* and *rrnL*) were located on the H strand, with lengths of 883 bp and 1393 bp, respectively. The lengths of 22 tRNA genes range from 65 (*trnQ*) to 74 bp (*trnK*), and all tRNA genes were able to fold into classic clover leaf structures.

**Figure 3.**
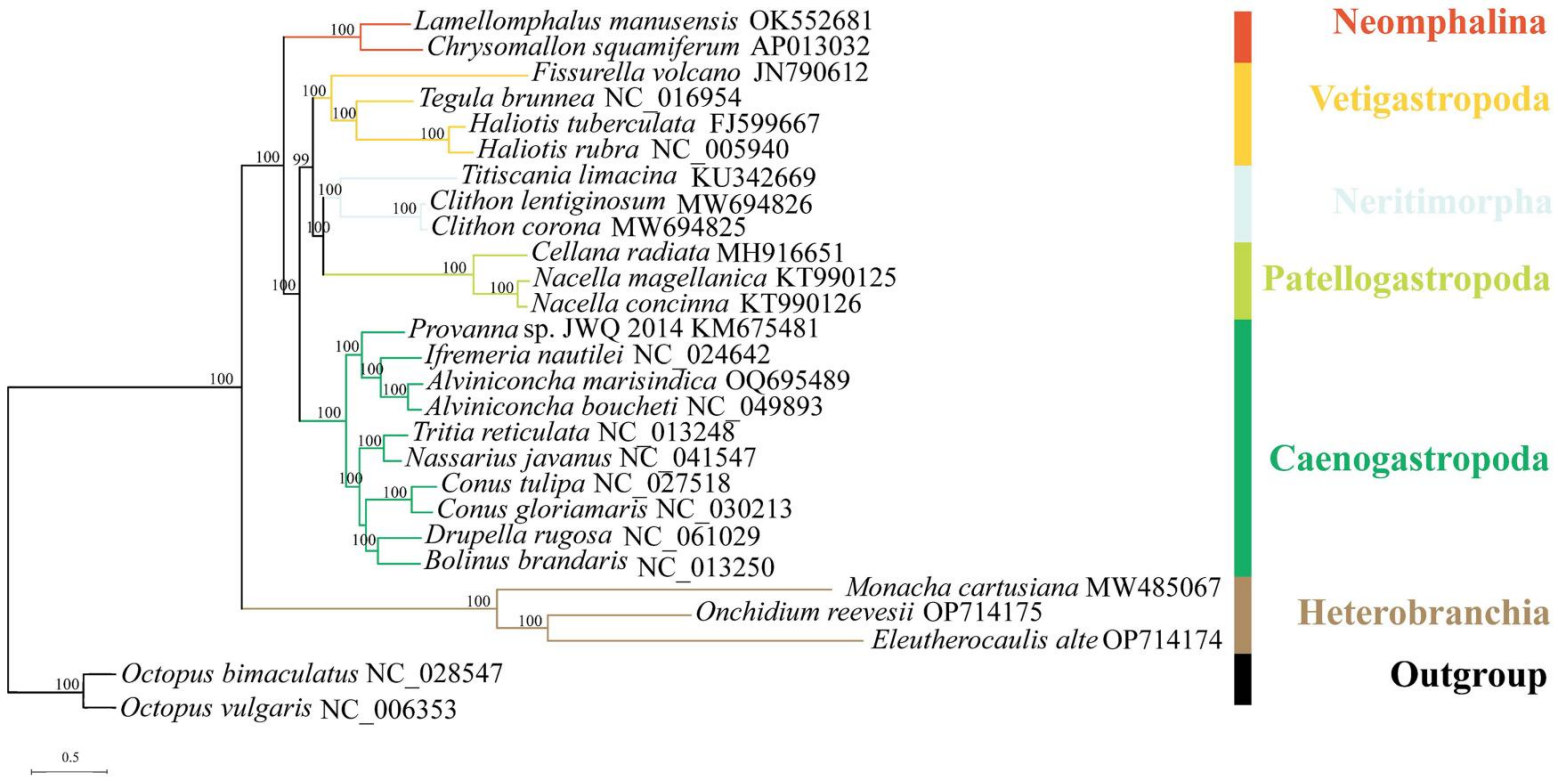
The maximum-likelihood (ML) phylogenetic tree for *A. marisindica* and the other Gastropoda species based on the concatenated nucleotide sequences of 13 protein-coding genes, and *A. marisindica* is placed within Caenogastropoda. Bootstrap support values are indicated at each node.

Based on the phylogenetic tree, *A. marisindica* was clustered in the Abyssochrysoidea clade and had the closest relationship with *A. boucheti* (Figure 3). In addition, the phylogenetic relationships among the six gastropod lineages were consistent with previous studies (Lee et al. 2019; Uribe et al. 2019). This research provides valuable mitogenomic data for further studies on the evolution of the deep-sea gastropods.

## Supplementary Material

Supplemental Material

## Data Availability

The genome sequence data that support the findings of this study are openly available in GenBank of NCBI at https://www.ncbi.nlm.nih.gov under the accession no. OQ695489. The associated Bio-Sample, BioProject, and SRA numbers are SAMN33865730, PRJNA947739, and SRR23970365, respectively.
