# Supplementary material for: The complete mitochondrial genome of the deep-sea paskentanid snail Alviniconcha marisindica (Caenogastropoda: abyssochrysoidea) from the Carlsberg Ridge
Source: Mitochondrial DNA B Resour. 2024 Jan 3;9(1):11–4. doi: 10.1080/23802359.2023.2298090 (PMC10769126; doi:10.1080/23802359.2023.2298090)
Supplement: Supplemental Material [file TMDN_A_2298090_SM5494.pdf]

Sequencing Depth and Coverage Map

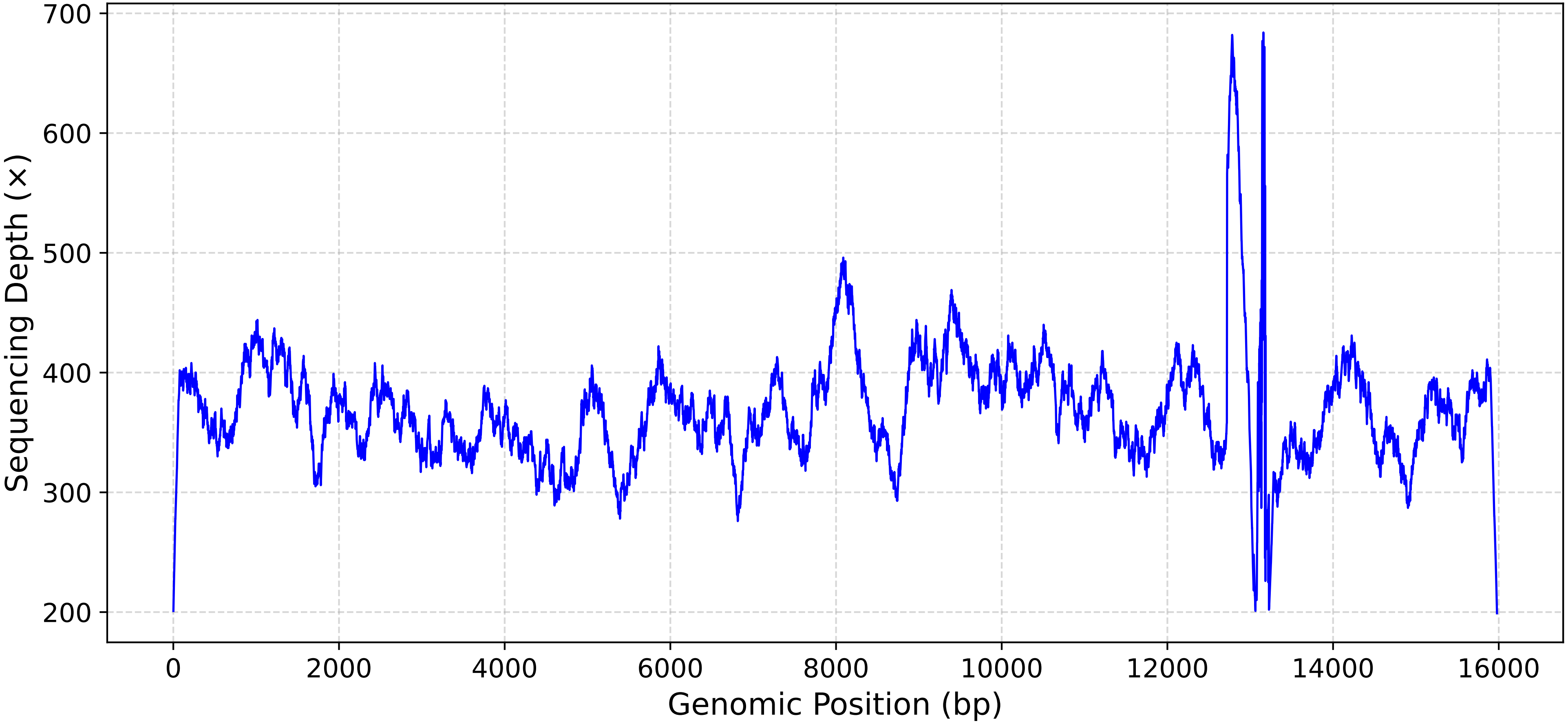

(1) Total genome length = 15,979 bp
(2) Average depth = 369.59 ×
(3) Maximal depth = 684 ×
(4) Minimal depth = 199 ×
